# A New Early Warning System for Predicting Adverse Outcomes in Hospitalized Patients With Neurological Conditions: A Retrospective Cohort Study

**DOI:** 10.7759/cureus.92068

**Published:** 2025-09-11

**Authors:** Carlos Arellano González, Luis Enrique Niembro Muñoz, Juan Jose Aguilar-Lugo-Gerez, Javier Sanchez Závala

**Affiliations:** 1 Internal Medicine, Médica Sur, Mexico City, MEX; 2 Internal Medicine, Instituto Nacional de Ciencias Médicas y Nutrición Salvador Zubirán, Mexico City, MEX

**Keywords:** early warning system, mortality, neurological patients, predictive accuracy, rapid response team

## Abstract

Introduction

Early Warning Scores (EWS) are widely used to identify acute clinical deterioration in hospitalized patients, particularly when integrated with rapid response teams (RRTs) as part of hospital safety systems. Although EWS have demonstrated benefits in reducing mortality and facilitating timely intensive care transfers, existing models have been primarily validated in general populations. Neurological and neurosurgical patients, despite being a significant subgroup of emergency admissions, are often underrepresented in these validation studies. Their unique clinical presentations - often subtle or atypical - pose additional challenges for early detection. This study aims to assess the predictive accuracy of various EWS for in-hospital mortality and urgent transfers among patients with neurological and neurosurgical diagnoses, and to develop a new, neurological-specific score (NEURO-EWS) tailored to this population.

Methods

This retrospective cohort study analyzed clinical records of adult patients with neurological conditions, divided into neurological and neurosurgical subgroups. The primary outcome was a composite of in-hospital mortality or urgent transfer to critical care; secondary outcomes analyzed these components separately. The predictive performance of EWS was evaluated at multiple time points using the area under the receiver operating characteristic curve (AUC), and calibration was assessed through probability plots. A novel scoring model, NEURO-EWS, was developed using multivariate logistic regression and validated in a separate cohort.

Results

Standard EWS showed lower predictive performance than expected. The modified Rapid Emergency Medicine Score (mREMS) achieved the highest accuracy among existing models (AUC: 0.68 in the overall cohort, 0.754 in neurological, and 0.646 in neurosurgical patients). The newly developed NEURO-EWS demonstrated superior performance in neurological patients (AUC: 0.782), outperforming all other scores.

Conclusion

Disease-specific EWS improves the detection of clinical deterioration in neurological patients. While mREMS remains the most reliable general model, NEURO-EWS provides enhanced predictive value for neurological populations and may support better clinical decision-making.

## Introduction

It is unacceptable for any hospital not to incorporate Early Warning Scores (EWS), alongside a rapid response team (RRT), within its quality assurance processes. Patients do not deteriorate suddenly; rather, we suddenly recognize their deterioration.

Neurological conditions rank as the third leading cause of admission to emergency services [1,2]. EWS were developed to identify patients experiencing acute clinical deterioration, enabling timely interventions to prevent adverse outcomes [3]. Numerous studies have highlighted the limited ability of healthcare centers to detect such deterioration in hospitalized patients, directly impacting prognosis. In response, the National Early Warning Score (NEWS) was introduced to standardize protocols for early identification of clinical deterioration and facilitate prompt therapeutic action [4].

EWS are designed based on theoretical models, physiological parameters, and pathophysiological mechanisms, and have been validated in general healthcare settings worldwide [5-9]. Typically, these scores incorporate vital signs (VS) and validated clinical scales, routinely used in hospitals - such as the Glasgow Coma Scale (GCS) - to detect acute health deterioration during hospitalization [1,9].

The implementation of EWS, in conjunction with RRTs, has rapidly improved early transfers to critical care units, reducing the need for invasive emergency procedures and lowering mortality rates [10]. Consequently, numerous EWS have been developed, many demonstrating significant predictive ability for cardiac arrest and mortality, thereby guiding critical care decisions [11]. Among the most widely validated and implemented are NEWS version 2 (NEWS2), the Rapid Acute Physiology Score (RAPS), and the modified Rapid Emergency Medicine Score (mREMS). NEWS2, originally developed for the English population, has been extensively validated internationally [5]; RAPS was developed and validated in the United States [6,7]; and mREMS originated in Thailand, with validations in multiple populations [12].

EWS have shown potential in predicting various adverse outcomes. However, most validation studies have involved general hospitalized populations, regardless of diagnosis or admission type, raising concerns about their applicability to specific groups [9,13-15]. To address this, recent studies have focused on specific populations [16-19]. NEWS2 was evaluated in patients with cardiovascular diseases in a retrospective cohort in London, reporting an area under the receiver operating characteristic curve (AUC) of 0.70 for predicting cardiac arrest. Additional variables that improved the score’s discriminative power were identified, such as age and heart rhythm, increasing the AUC above 0.90 [20].

Despite the proliferation of EWS and recent context-specific validations, data on their performance in neurological patients remain scarce and are mainly limited to cerebrovascular disease [16,18,19], traumatic brain injury [17], or emergency department settings [1]. The detection of clinical deterioration in neurological patients is particularly challenging due to negative symptomatology [2]. Although current results are modest, incorporating additional variables may improve predictive accuracy, and evaluating alternative EWS for neurological patients is warranted. To date, no EWS has been specifically developed for hospitalized neurological patients.

The primary objective of this study is to evaluate the predictive capacity of various EWS (NEWS2, mREMS, and RAPS) for mortality, critical care transfer, or a composite outcome in neurological and neurosurgical patients in the hospital setting. The composite outcome is defined as the sum of mortality and critical care transfer events, with the aim of analyzing them as a whole, since either event directly impacts patient prognosis during hospitalization. Considering these outcomes separately, while informative, falls short of the main purpose of an effective EWS. For instance, if an EWS is effective at detecting mortality, but no intervention is undertaken to prevent it, its utility is limited. Conversely, if an EWS accurately predicts critical care transfer, but mortality ultimately remains unchanged, its impact is also insufficient.

The secondary objective of this work is to analyze variables associated with in-hospital mortality or urgent critical care transfer, and to identify those with the greatest impact. Based on these findings, we plan to develop a novel predictive model, incorporating these variables, to enhance predictive accuracy for mortality, critical care transfer, or the previously defined composite outcome in neurological patients.

Finally, by achieving these two objectives, we aim to fulfill a third objective: to compare the predictive capacity of the new NEURO-EWS (Neurological Early Warning Score) model against the other EWS previously analyzed. Previous research supports the idea that adding relevant variables, such as other demographic or biochemical characteristics (e.g., interleukin levels or copeptin levels), can substantially improve the performance of existing EWS [21,22]. Therefore, this may lead to a greater impact on patient prognosis and a reduction in mortality.

To define our population, we selected the 14 most common neurological diagnoses worldwide for detailed analysis [23], aiming to identify the most suitable EWS for this patient group.

## Materials and methods

Study design

We conducted a retrospective cohort study at a tertiary care center in Mexico City, spanning September 1, 2019, through December 31, 2022. The study protocol received approval from the Ethics and Research Committee of Hospital Médica Sur, Mexico City, Mexico (committee register: CONBIOÉTICA-09-CEI-018-20160729), with approval reference number 2024-EXT-810. We included all consecutive adult patients who met the eligibility criteria during the study period, yielding a complete cohort for analysis.

Study population

The required sample size was estimated at 597 patients, calculated using the formula $n = \frac{Z^2 S^2}{d^2}$ to achieve 80% statistical power. Eligible patients were adults (≥18 years) who were inpatients, having been admitted through the Emergency Department, direct admission, or the operating room. Inclusion diagnoses encompassed cerebrovascular disease, demyelinating diseases, traumatic brain injury, headaches, neuropathies, metabolic encephalopathies, epilepsy, dementias, movement disorders, Gamma Knife radiosurgery (malignant and non-malignant), craniotomy, spine surgery, and pituitary adenoma.

**Figure 1:**
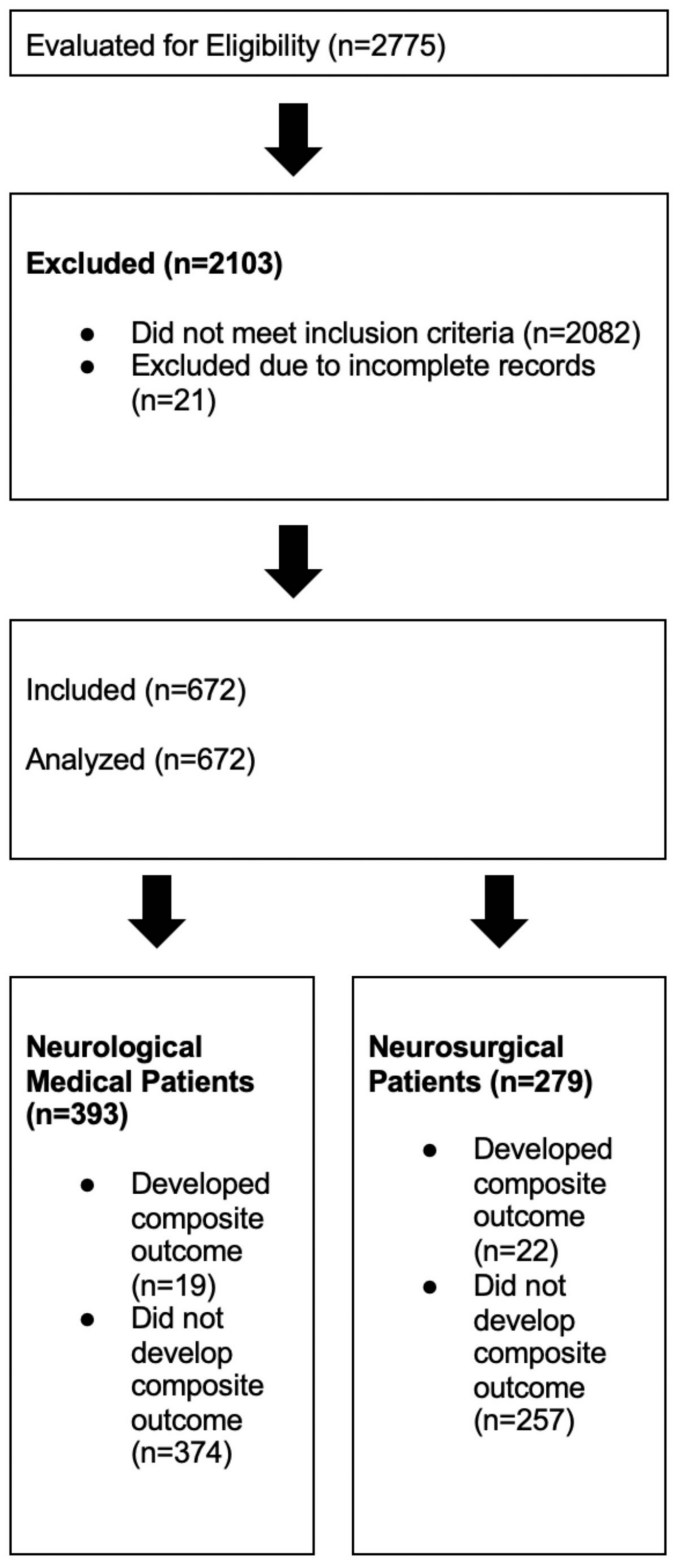
STROBE Diagram

Records lacking admission or discharge documentation, or with incomplete critical data - such as medical record number, full name, admission and discharge dates, diagnosis, sex, age, GCS at admission and within 24 hours prior to discharge/transfer/death, or NEWS2 at admission and within 24 hours prior to discharge/transfer/death - were excluded. A detailed patient selection flowchart is shown in Figure 1.

Outcomes

The primary outcome was a composite of in-hospital mortality and urgent transfer to a critical care unit, where “urgent transfer” is understood as the imminent need to move the patient to the critical care area due to the risk of death if the transfer is not performed, regardless of time or other factors. Each component was also assessed individually as secondary outcomes.

Data collection

Clinical records were retrospectively reviewed to extract relevant outcomes and variables. Demographic, clinical, and anthropometric data were collected alongside VS, peripheral oxygen saturation (SpO₂), oxygen supplementation status, and GCS scores at hospital admission. Using these data, NEWS2, mREMS, and RAPS were calculated; all of these scales are open access [4,12,17].

To construct the model that predicts outcomes within the subsequent eight hours, physiological variables and EWS values for patients who developed an outcome (“cases”) were extracted from the time window within eight hours prior to the event. For patients who did not develop an outcome (“controls”), we extracted the single most abnormal value observed during the first 48 hours from hospital admission to serve as a comparator. After exclusions for incomplete records, there were no missing data in the analytic dataset for variables used in the models; therefore, no imputation was required.

Statistical analysis

All analyses were performed using IBM SPSS Statistics for Windows, Version 24 (Released 2016; IBM Corp., Armonk, NY, USA). Two prespecified multivariable logistic regression models were developed: (i) a hospitalization model to predict the occurrence of the composite outcome (in-hospital mortality or urgent transfer to critical care) at any time during the index hospitalization using admission measurements, and (ii) an eight-hour model to predict the composite outcome within the next eight hours using the time-window definitions described above. Candidate predictors included age, sex, body mass index, surgical status, systolic and diastolic blood pressure, mean arterial pressure, heart rate, respiratory rate, temperature, SpO₂, supplemental oxygen requirement, and GCS. Continuous predictors were modeled both linearly and as categories informed by observed probability distributions. Variables with Wald test p < 0.20 in either modeling approach were retained for multivariable analyses.

To derive the new score (NEURO-EWS), we included predictors that were statistically significant in either of the two logistic models. Complete specifications of the two original logistic regression models (predictors, coefficients, and intercepts) are provided in the Appendix. Regression β-coefficients from the final models were scaled and rounded to the nearest integer to assign item weights and generate the point-based score. Discrimination was quantified by the AUC, with 95% confidence intervals, calculated at admission and within eight hours prior to the event. Calibration was assessed by plotting observed outcome probabilities across score categories.

We categorized the total NEURO-EWS by visually inspecting the observed probability of the composite outcome at each possible score in the derivation cohort and selecting cut-points at the inflection transitions; adjacent scores with similar risks were collapsed to ensure a monotonic gradient and adequate counts per stratum. The resulting thresholds (minimal ≤1, low 2-5, intermediate 6, high ≥7) were prespecified and applied unchanged to the validation cohort.

For internal validation, the dataset was randomly partitioned a priori into a derivation (75%) and validation (25%) cohort, stratified by surgical status and outcome. All head-to-head comparisons between NEURO-EWS and existing EWS (NEWS2, mREMS, RAPS, GCS) were performed in the validation cohort to avoid optimism bias.

Differences in outcome probabilities between score categories were evaluated with the Mann-Whitney U test. Statistical significance was set at two-sided p < 0.05.

Development of a novel predictive model

The dataset was randomly partitioned into derivation (75%) and validation (25%) cohorts, stratified by surgical status and outcome. Multivariate logistic regression identified physiological variables associated with clinical outcomes.

Candidate predictors included age, sex, body mass index, surgical intervention during the current admission, systolic and diastolic blood pressure, mean arterial pressure, heart rate, respiratory rate, temperature, SpO₂, supplemental oxygen requirement, and GCS score. Models were developed using parameters recorded at admission and again using data from eight hours prior to the critical event.

**Figure 2:**
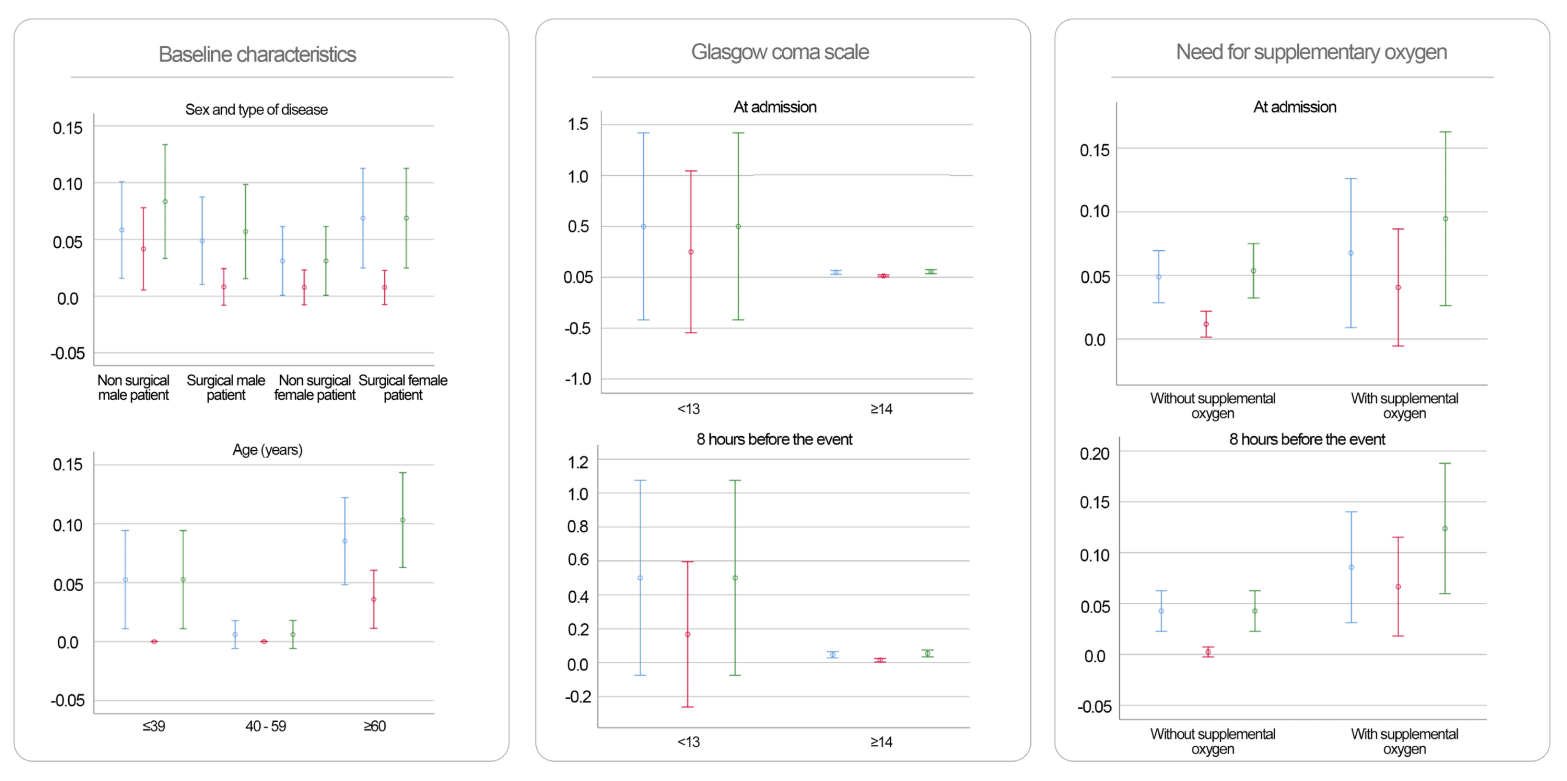
Predictors Assessed for the Development of NEURO-EWS (Baseline Characteristics, Glasgow Coma Scale, and Need for Supplementary Oxygen) This figure presents the predictors analyzed for the development of NEURO-EWS. It illustrates the distribution of the evaluated outcomes across the predictive variables, which are categorized based on the probability distribution analysis. Color coding of outcomes: blue = urgent transfer to the critical care area, red = death, green = composite outcome (urgent transfer to the critical care area or death). NEURO-EWS: Neurological Early Warning Score

**Figure 3:**
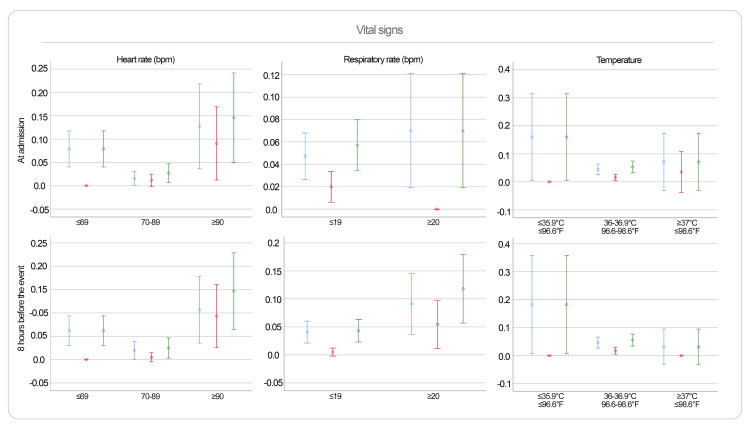
Predictors Assessed for the Development of NEURO-EWS (Heart Rate, Respiratory Rate, and Temperature) This figure presents the predictors analyzed for the development of NEURO-EWS. It illustrates the distribution of the evaluated outcomes across the predictive variables, which are categorized based on the probability distribution analysis. Color coding of outcomes: blue = urgent transfer to the critical care area, red = death, green = composite outcome (urgent transfer to the critical care area or death). NEURO-EWS: Neurological Early Warning Score; bpm: Beats per Minute

Continuous variables were modeled both linearly and categorically. Predictors with Wald test p-values < 0.2 in either model were retained. Logistic regression β-coefficients were rounded to assign weights in the new scoring system. The predictors evaluated and their associations with outcomes are illustrated in Figures 2-3.

Discriminative ability of the new score was assessed by AUC at admission and eight hours prior to the critical event, stratified by surgical status and outcome.

## Results

Sample characteristics

A total of 671 electronic medical records meeting the predefined diagnostic criteria were included, as shown in Table 1. Of these, 340 patients (50.6%) had neurosurgical diagnoses. The median age was 56 years (interquartile range (IQR), 42-69 years), with a nearly equal sex distribution and a slight female predominance (51.2%).

**Table 1:** Demographic Characteristics Demographic characteristics of patients: dichotomous variables are presented as n (%), and numerical variables as median (IQR). BMI: Body Mass Index; BPM: Beats Per Minute; DBP: Diastolic Blood Pressure; GCS: Glasgow Coma Score; HR: Heart Rate; IQR: Interquartile Range; kg: Kilograms; m²: Square Meter; mmHg: Millimeters of Mercury; mREMS: Modified Rapid Emergency Medicine Score; NEWS2: National Early Warning Score; O₂: Oxygen; RAPS: Rapid Acute Physiology Score; RPM: Respiration Per Minute; RR: Respiratory Rate; SBP: Systolic Blood Pressure; SpO₂: Peripheral Oxygen Saturation

Variable	Median (IQR) or n (%)
Age	56 (42-69)
Sex (female)	343 (51.2%)
Diagnosis (surgical)	340 (50.6%)
BMI (kg/m²)	24.9 (22.5-28.1)
In-hospital mortality	13 (1.9%)
Urgent transfer to critical care	35 (5.2%)
Composite outcome	41 (6.1%)
SBP at admission (mmHg)	118 (106-131)
SBP 8 hours before outcome (mmHg)	104 (94-123)
DBP at admission (mmHg)	72 (65-79)
DBP 8 hours before outcome (mmHg)	65 (59-74)
HR at admission (bpm)	74 (65-83)
HR 8 hours before outcome (bpm)	72 (64-83)
RR at admission (rpm)	18 (18-19)
RR 8 hours before outcome (rpm)	18 (18-19)
Temperature at admission (°C)	36.4 (36.2-36.6)
Temperature 8 hours before outcome (°C)	36.4 (36.2-36.6)
Need for supplemental O_2_ at admission	98 (14.6%)
Need for supplemental O_2_ 8 hours before outcome	138 (20.5%)
SpO_2_ at admission (%)	94 (93-96)
SpO_2_ 8 hours before outcome (%)	92 (91-95)
GCS at admission	15 (15-15)
GCS 8 hours before outcome	15 (15-15)
NEWS-2 at admission	2 (1-3)
NEWS-2 8 hours before outcome	4 (3-5)
mREMS at admission	3 (1-4)
mREMS 8 hours before outcome	3 (2-5)
RAPS at admission	0 (0-2)
RAPS 8 hours before outcome	2 (0-2)

Among patients with neurological conditions, cerebrovascular disease was the most prevalent diagnosis (117 patients, 17.4%), while spine surgery was the most frequent diagnosis among neurosurgical patients (102 patients, 15.2%). A complete list of diagnoses and their frequencies is available in Table 2. There were 41 composite outcomes (6.1%) observed, comprising 35 urgent transfers to critical care units and 13 in-hospital deaths.

**Table 2:** Neurological Diagnoses and Their Frequencies

Diagnosis	n (%)
Cerebrovascular event	117 (17.4)
Spinal surgery	102 (15.2)
Metabolic encephalopathies	76 (11.3)
Craniotomy	67 (10.0)
Gamma Knife surgery for malignant disease	64 (9.5)
Epilepsy	51 (7.6)
Traumatic brain injury	40 (6.0)
Headaches	36 (5.4)
Neuropathies	33 (4.9)
Demyelinating disease	25 (3.7)
Gamma Knife surgery for benign disease	25 (3.7)
Pituitary adenoma	21 (3.1)
Dementias	10 (1.5)
Movement disorders	5 (0.7)

Discriminative performance of EWS 

All EWS demonstrated lower discriminative performance than what has been reported in the international literature. Additionally, EWS were generally less effective in patients with neurosurgical conditions compared to those with medical neurological diagnoses.

The NEWS2 score exhibited the highest AUC for predicting in-hospital mortality - both at admission and within the subsequent eight hours - across surgical, non-surgical, and combined patient cohorts. Conversely, the mREMS score yielded the highest AUCs for predicting critical care admission and the composite outcome, both at admission and within the following eight hours, in all patient subgroups. In contrast, the RAPS and GCS scales generally did not discriminate between patients who would or would not experience adverse outcomes better than chance.

**Figure 4:**
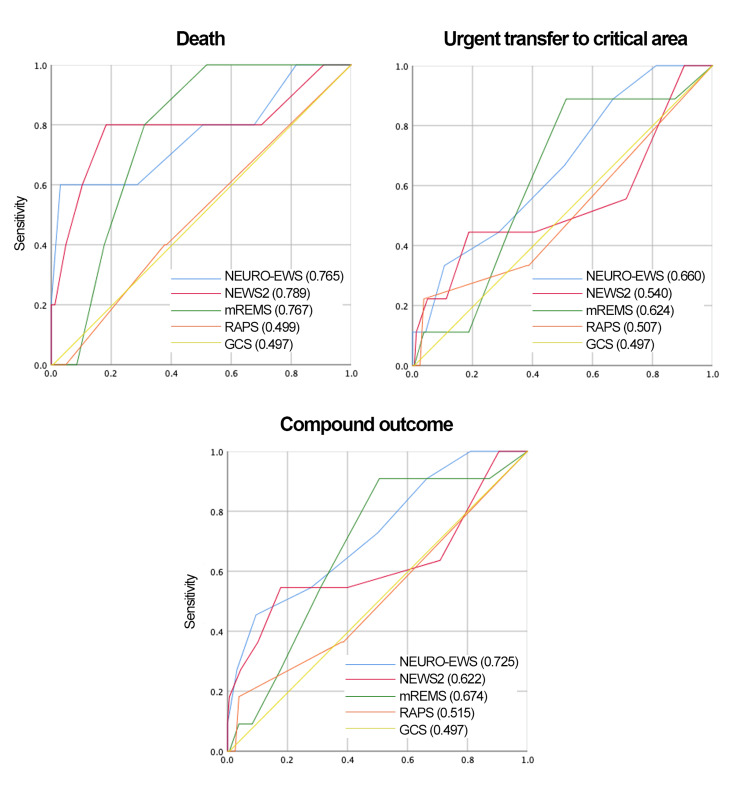
ROC Curves for 4 EWS and NEURO-EWS for the Compound Outcome and Secondary Outcomes in Neurological and Neurosurgical Patients at 8 Hours Prior to the Outcome The areas under the ROC curve, for the 4 EWS analyzed and the NEURO-EWS, within the parentheses of each graphic, is the numerical value of the AUC. GCS: Glasgow Coma Score, mREMS: Modified Rapid Emergency Medicine Score, NEWS-2: National Early Warning Score, RAPS: Rapid Acute Physiology Score; ROC: Receiver Operating Characteristic; AUC: Area Under the Curve; EWS: Early Warning Score; NEURO-EWS: Neurological Early Warning Score

**Figure 5:**
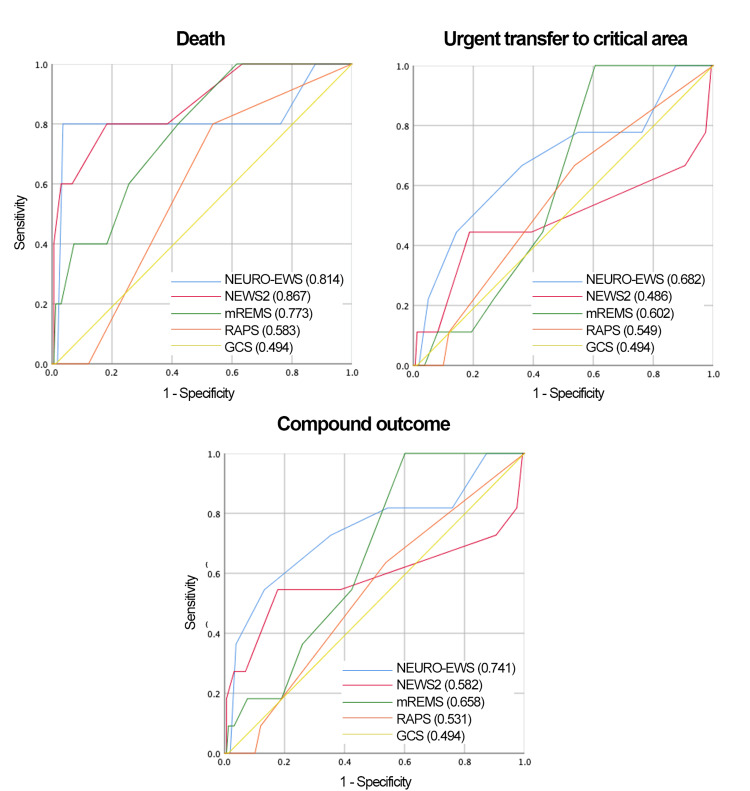
ROC Curve for 4 EWS and NEURO-EWS for the Compound Outcome and Secondary Outcomes Studied in Neurological Patients and Neurosurgical Patients at 8 Hours Prior to Outcome The areas under the ROC curve, for the 4 EWS analyzed and the NEURO-EWS, within the parentheses of each graphic, is the numerical value of the AUC. GCS: Glasgow Coma Score, mREMS: Modified Rapid Emergency Medicine Score, NEWS-2: National Early Warning Score, RAPS: Rapid Acute Physiology Score; ROC: Receiver Operating Characteristic; AUC: Area Under the Curve; EWS: Early Warning Score; NEURO-EWS: Neurological Early Warning Score

Receiver operating characteristic (ROC) curves for all EWS, including the newly developed model, are presented in Figures 4-5.

The discriminative performance of all scoring systems in the full sample is summarized in Table 3.

**Table 3:** Area Under the ROC Curve in Patients With Both Surgical and Non-surgical Neurological Conditions The areas under the ROC curve are shown, with 95% confidence intervals (CIs) in parentheses. Tests that predict significantly better than random (i.e., the 95% CI does not cross 0.5) are shown in bold. GCS: Glasgow Coma Score, mREMS: Modified Rapid Emergency Medicine Score, NEWS-2: National Early Warning Score, RAPS: Rapid Acute Physiology Score; ROC: Receiver Operating Characteristic; NEURO-EWS: Neurological Early Warning Score

Early warning system	In-hospital mortality	Urgent transfer to a critical care unit	Composite outcome
Initial NEURO-EWS	0.765 (0.502-1)	0.66 (0.488-0.832)	0.725 (0.566-0.884)
Initial NEWS2	0.789 (0.522-1)	0.54 (0.309-0.771)	0.622 (0.408-0.836)
Initial mREMS	0.767 (0.655-0.879)	0.624 (0.459-0.789)	0.674 (0.527-0.821)
Initial RAPS	0.499 (0.25-0.748)	0.507 (0.291-0.723)	0.515 (0.323-0.707)
Initial GCS	0.497 (0.24-0.754)	0.497 (0.303-0.691)	0.497 (0.321-0.673)
8h prior NEURO-EWS	0.832 (0.569-1)	0.723 (0.325-1)	0.832 (0.569-1)
8h prior NEWS2	0.853 (0.673-1)	0.756 (0.497-1)	0.853 (0.673-1)
8h prior mREMS	0.782 (0.611-0.953)	0.696 (0.473-0.919)	0.782 (0.611-0.953)
8h prior RAPS	0.579 (0.375-0.783)	0.669 (0.506-0.832)	0.579 (0.375-0.783)
8h prior GCS	0.494 (0.235-0.753)	0.494 (0.163-0.825)	0.494 (0.235-0.753)

Discrimination data for non-surgical and surgical subgroups are provided in Tables 4-5, respectively. 

**Table 4:** Area Under the ROC Curve in Patients With Non-surgical Neurological Conditions The areas under the ROC curve are shown, with 95% confidence intervals (CIs) in parentheses. Tests that predict significantly better than random (i.e., the 95% CI does not cross 0.5) are shown in bold. GCS: Glasgow Coma Score, mREMS: Modified Rapid Emergency Medicine Score, NEWS-2: National Early Warning Score, RAPS: Rapid Acute Physiology Score; ROC: Receiver Operating Characteristic; NEURO-EWS: Neurological Early Warning Score

Early warning system	In-hospital mortality	Urgent transfer to a critical care unit	Composite outcome
Initial NEURO-EWS	0.787 (0.534-1)	0.638 (0.299-0.977)	0.787 (0.534-1)
Initial NEWS2	0.806 (0.547-1)	0.685 (0.307-1)	0.806 (0.547-1)
Initial mREMS	0.773 (0.653-0.893)	0.69 (0.561-0.819)	0.773 (0.653-0.893)
Initial RAPS	0.522 (0.261-0.783)	0.488 (0.161-0.815)	0.522 (0.261-0.783)
Initial GCS	0.5 (0.237-0.763)	0.5 (0.165-0.835)	0.5 (0.237-0.763)
8h prior NEURO-EWS	0.832 (0.569-1)	0.723 (0.325-1)	0.832 (0.569-1)
8h prior NEWS2	0.853 (0.673-1)	0.756 (0.497-1)	0.853 (0.673-1)
8h prior mREMS	0.782 (0.611-0.953)	0.696 (0.473-0.919)	0.782 (0.611-0.953)
8h prior RAPS	0.579 (0.375-0.783)	0.669 (0.506-0.832)	0.579 (0.375-0.783)
8h prior GCS	0.494 (0.235-0.753)	0.494 (0.163-0.825)	0.494 (0.235-0.753)

**Table 5:** Area Under the ROC Curve in Patients With Surgical Neurological Conditions The areas under the ROC curve are shown, with 95% confidence intervals (CIs) in parentheses. It was not possible to plot ROC curves for the in-hospital mortality outcome as no cases were presented in the validation subgroup. GCS: Glasgow Coma Score, mREMS: Modified Rapid Emergency Medicine Score, NEWS-2: National Early Warning Score, RAPS: Rapid Acute Physiology Score; ROC: Receiver Operating Characteristic; NEURO-EWS: Neurological Early Warning Score

Early warning system	Urgent transfer to a critical care unit	Composite outcome
Initial NEURO-EWS	0.657 (0.467-0.847)	0.657 (0.467-0.847)
Initial NEWS2	0.448 (0.164-0.732)	0.448 (0.164-0.732)
Initial mREMS	0.582 (0.347-0.817)	0.582 (0.347-0.817)
Initial RAPS	0.51 (0.23-0.79)	0.51 (0.23-0.79)
Initial GCS	0.494 (0.255-0.733)	0.494 (0.255-0.733)
8h prior NEURO-EWS	0.642 (0.417-0.867)	0.642 (0.417-0.867)
8h prior NEWS2	0.346 (0.021-0.671)	0.346 (0.021-0.671)
8h prior mREMS	0.547 (0.41-0.684)	0.547 (0.41-0.684)
8h prior RAPS	0.489 (0.246-0.732)	0.489 (0.246-0.732)
8h prior GCS	0.494 (0.255-0.733)	0.494 (0.255-0.733)

**Figure 6:**
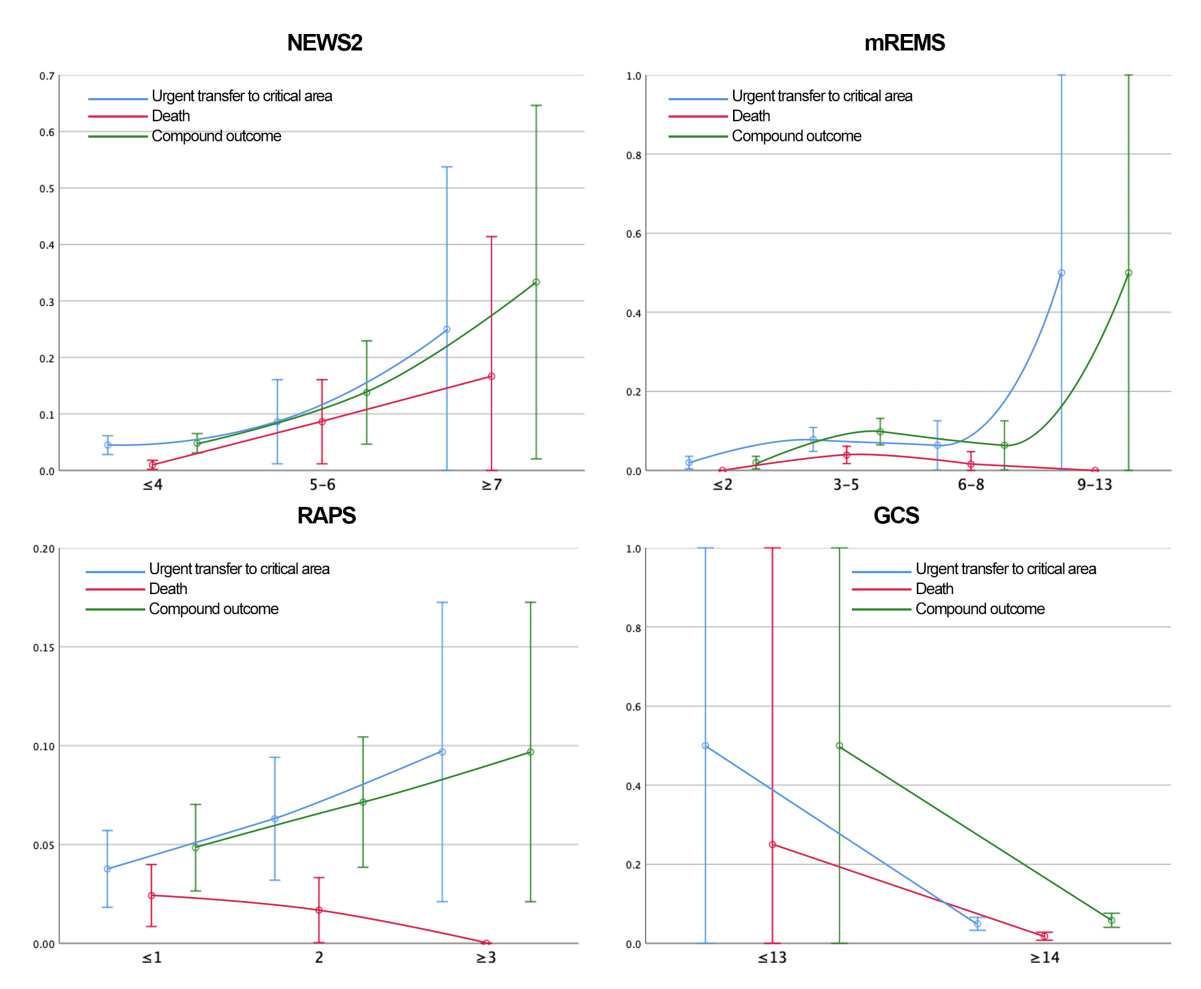
Distribution of the Probability of the Outcomes Studied by Category of the Scales Analyzed at Admission GCS: Glasgow Coma Score; mREMS: Modified Rapid Emergency Medicine Score; NEWS-2: National Early Warning Score; RAPS: Rapid Acute Physiology Score

**Figure 7:**
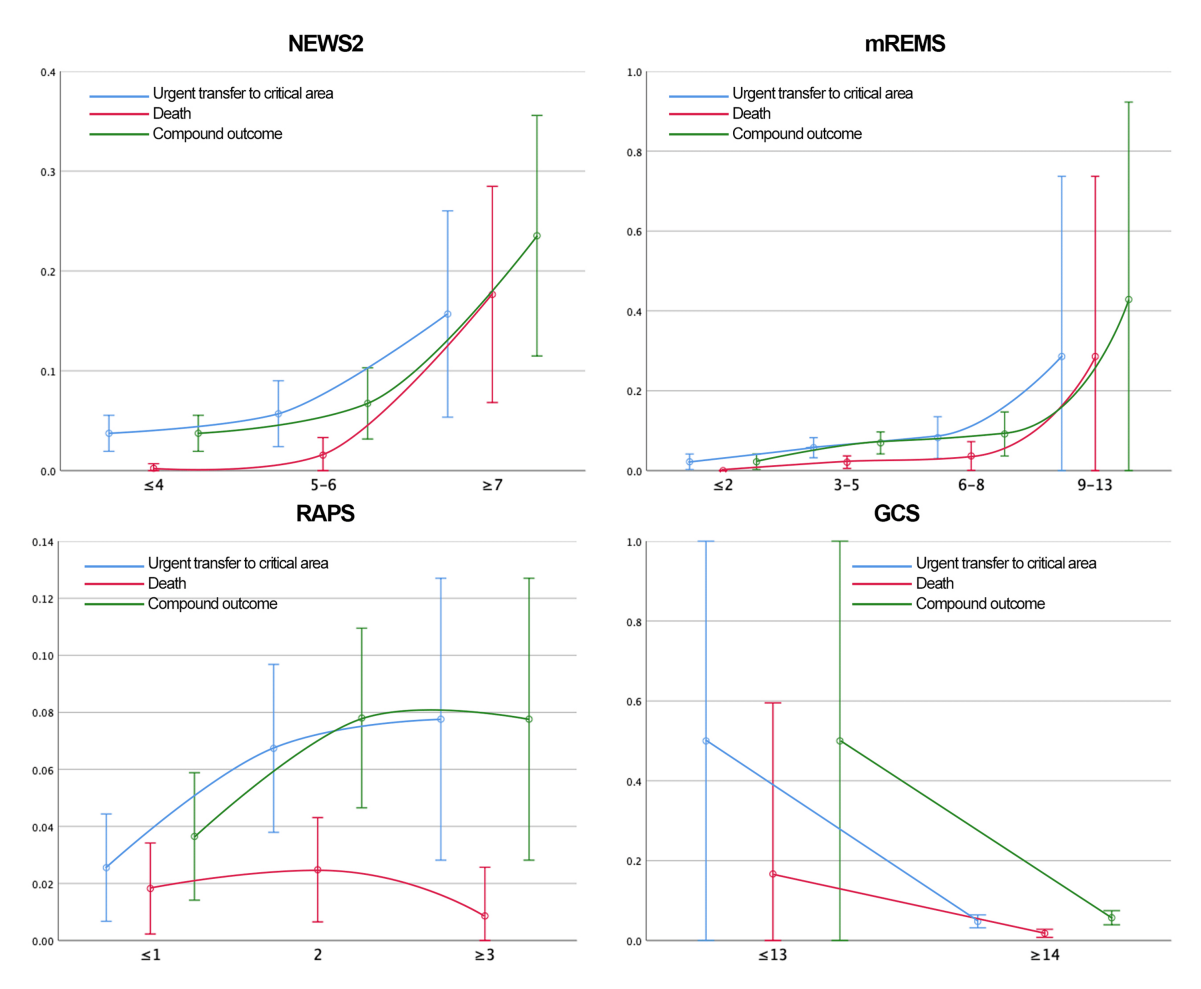
Distribution of the Probability of the Outcomes Studied by Category of the Scales Analyzed at 8 Hours Prior to Outcome GCS: Glasgow Coma Score; mREMS: Modified Rapid Emergency Medicine Score; NEWS-2: National Early Warning Score; RAPS: Rapid Acute Physiology Score

Probability analyses for each outcome by EWS category are presented in Tables 6-9 and Figures 6-7.

**Table 6:** Probability of Studied Outcomes by NEWS-2 Categories Highlighted Mann-Whitney U values indicate differences between categories with a p-value < 0.05. NEWS-2: National Early Warning Score

Outcome	NEWS-2, ≤4	NEWS-2, 5-6	NEWS-2, ≥7	p-value
In-hospital mortality at hospital admission	1% (0.2-1.8%)	8.6% (1.3-15.9%)	16.7% (0-38.7%)	1.56 x 10^-7^
Urgent transfer to a critical care unit at hospital admission	4.5% (2.8-6.1%)	8.6% (1.3-15.9%)	25% (0-50.6%)	0.010261
Composite outcome at hospital admission	4.8% (3.1-6.5%)	13.8% (4.8-22.7%)	33.3% (5.5-61.2%)	0.000029
In-hospital mortality 8h prior to outcome	0.2% (0-0.7%)	1.6% (0-3.3%)	17.6% (7.1-28.2%)	3.4917 x 10^-8^
Urgent transfer to a critical care unit 8h prior to outcome	3.7% (1.9-5.5%)	5.7% (2.4-9%)	15.7% (5.6-25.8%)	0.005945
Composite outcome 8h prior to outcome	3.7% (1.9-5.5%)	6.7% (3.2-10.3%)	23.5% (11.8-35.3%)	0.000035

**Table 7:** Probability of Studied Outcomes by mREMS Categories Highlighted Mann-Whitney U values indicate differences between categories with a p-value < 0.05. mREMS: Modified Rapid Emergency Medicine Score

Outcome	mREMS, ≤2	mREMS, 3-5	mREMS, 6-8	mREMS, ≥9	p-value
In-hospital mortality at hospital admission	0% (0-0%)	3.9% (1.7-6.1%)	1.6% (0-4.7%)	0% (0-0%)	0.006025
Urgent transfer to a critical care unit at hospital admission	2% (0.4-3.6%)	7.8% (4.8-10.9%)	6.3% (0.3-12.4%)	50% (0-100%)	0.001223
Composite outcome at hospital admission	6% (0-17.8%)	9.8% (6.5-13.1%)	6.3% (0.3-12.4%)	50% (0-100%)	0.000259
In-hospital mortality 8h prior to outcome	0% (0-0%)	2.1% (0.6-3.7%)	3.7% (0.1-7.2%)	28.6% (0-64.7%)	0.000729
Urgent transfer to a critical care unit 8h prior to outcome	2.2% (0.3-4.2%)	5.7% (3.2-8.2%)	8.3% (3.1-13.4%)	28.6% (0-64.7%)	0.002866
Composite outcome 8h prior to outcome	2.2% (0.3-4.2%)	6.9% (4.2-9.7%)	9.2% (3.7-14.6%)	42.9% (3.3-82.5%)	0.000438

**Table 8:** Probability of Studied Outcomes by RAPS Categories Highlighted Mann-Whitney U values indicate differences between categories with a p-value < 0.05. RAPS: Rapid Acute Physiology Score

Outcome	RAPS, ≤1	RAPS, 2	RAPS, ≥3	p-value
In-hospital mortality at hospital admission	2.4% (0.9-4%)	1.7% (0-3.3%)	0% (0-0%)	0.234621
Urgent transfer to a critical care unit at hospital admission	3.8% (1.8-5.7%)	6.3% (3.2-9.4%)	9.7% (2.3-17.1%)	0.038077
Composite outcome at hospital admission	4.8% (2.7-7%)	7.1% (3.9-10.4%)	9.7% (2.3-17.1%)	0.098199
In-hospital mortality 8h prior to outcome	1.8% (0.2-3.4%)	2.5% (0.7-4.3%)	0.9% (0-2.6%)	0.797835
Urgent transfer to a critical care unit 8h prior to outcome	2.6% (0.7-4.4%)	6.7% (3.8-9.7%)	7.8% (2.9-12.6%)	0.011505
Composite outcome 8h prior to outcome	3.6% (1.4-5.9%)	7.8% (4.7-10.9%)	7.8% (2.9-12.6%)	0.043148

**Table 9:** Probability of Studied Outcomes by GCS Categories Highlighted Mann-Whitney U values indicate differences between categories with a p-value < 0.05. GCS: Glasgow Coma Score

Outcome	GCS, ≤13	GCS, ≥14	p-value
In-hospital mortality at hospital admission	25% (0-74%)	1.8% (0.8-2.8%)	0.028341
Urgent transfer to a critical care unit at hospital admission	50% (0-100%)	4.9% (3.3-6.6%)	0.010577
Composite outcome at hospital admission	50% (0-100%)	5.8% (4.1-7.6%)	0.023386
In-hospital mortality 8h prior to outcome	16.7% (0-49.3%)	1.8% (0.8-2.8%)	0.126044
Urgent transfer to a critical care unit 8h prior to outcome	50% (6.2-93.8%)	4.8% (3.2-6.4%)	0.00312
Composite outcome 8h prior to outcome	50% (6.2-93.8%)	5.7% (3.9-7.5%)	0.009106

Calibration analysis

A linear relationship was observed between the NEWS2 score at admission and the incidence of all three outcomes throughout hospitalization. However, when predicting outcomes within eight hours, the relationship with the NEWS2 score was exponential.

The mREMS score exhibited exponential relationships with outcomes at both time points, except for in-hospital mortality prediction at admission, where higher scores paradoxically correlated with a lower likelihood of death. For the RAPS score, a direct linear relationship was noted with critical care admission and the composite outcome, while an inverse linear relationship was found with in-hospital mortality during the hospital stay. Within the eight-hour prediction window, RAPS demonstrated a logarithmic trend, with little differentiation between medium- and high-score categories.

GCS scores were broadly associated with the likelihood of all outcomes; however, due to limited variability in score distribution, scores were dichotomized, precluding the identification of a clear gradient of risk.

Development of a novel predictive model

**Figure 8:**
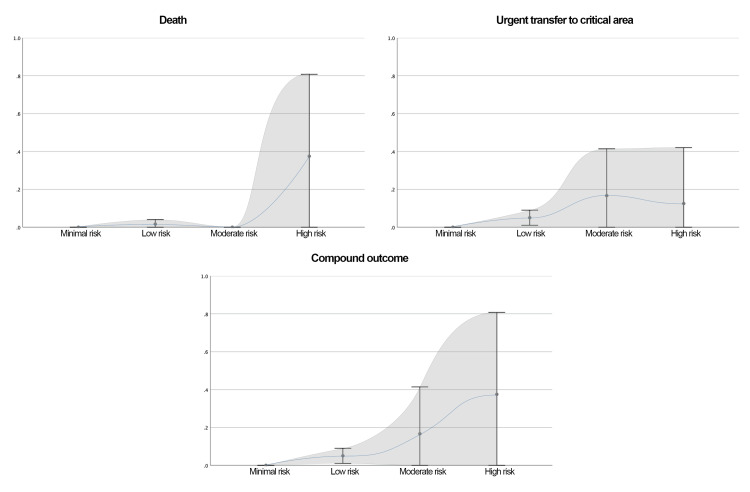
Distribution of the Probability of the Outcomes Studied by Category of NEURO-EWS at Admission The calibration plot illustrates the probability of the studied outcomes according to the categories defined by the new scale. The risk categories are as follows: minimal risk, ≤1 point; low risk, 2-5 points; intermediate risk, 6 points; and high risk, ≥7 points The shaded gray area represents the 95% confidence interval (95% CI). NEURO-EWS: Neurological Early Warning Score

**Figure 9:**
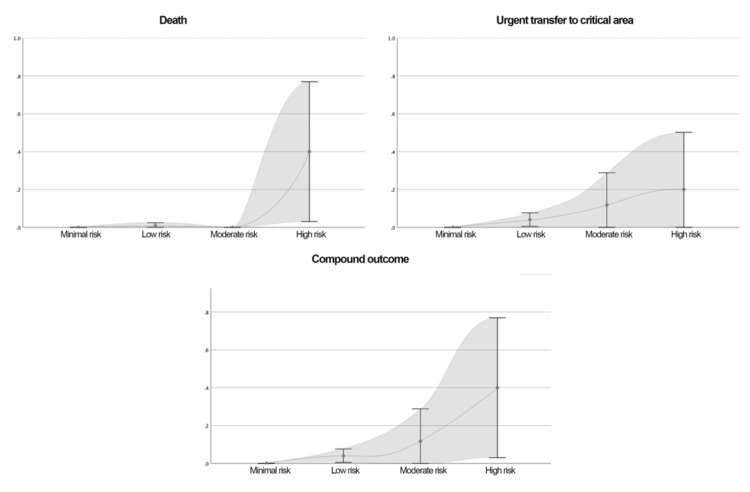
Distribution of the Probability of the Outcomes Studied by Category of NEURO-EWS at 8 Hours Prior to Outcome The calibration plot illustrates the probability of the studied outcomes according to the categories defined by the new scale. The risk categories are as follows: minimal risk, ≤1 point; low risk, 2-5 points; intermediate risk, 6 points; and high risk, ≥7 points. The shaded gray area represents the 95% confidence interval (95% CI). NEURO-EWS: Neurological Early Warning Score

The newly developed predictive model is presented in Table 10. This model outperformed NEWS2, mREMS, RAPS, and GCS in predicting the composite outcome, both at hospital admission and eight hours before a critical event. AUC values and ROC curves for the new model are shown in Table 3 and Figures 4-5, respectively. Calibration plots are shown in Figures 8-9.

**Table 10:** Scores of NEURO-EWS to Predict Adverse Outcomes in Patients With Neurological Conditions Points assigned to the different values in NEURO-EWS to predict adverse outcomes in patients with neurological conditions. Bpm: Beats per Minute; GCS: Glasgow Coma Score; O_2_: Oxygen; Rpm: Respiration per Minute; NEURO-EWS: Neurological Early Warning Score

Predictor	Patient clinical characteristics					
Assigned points	2	1	0	1	2	3
Type of patient	-	-	Women without surgical condition	All other patients	-	-
Age (years)	≤39	-	40-59	-	-	≥60
Heart rate (bpm)	-	≤69	70-89	-	≥90	-
Respiratory rate (rpm)	-	-	≤19	≥20	-	-
Temperature (°C)	-	≤35.9	≥36.0	-	-	-
Need for supplemental O_2_	-	-	No	Yes	-	-
GCS	≤13	-	≥14	-	-	-

Outcome probabilities across score categories are provided in Table 11.

**Table 11:** Probability of Different Outcomes by Categories of NEURO-EWS Mann-Whitney U: Differences between categories with a p-value < 0.05 are shown in bold. NEURO-EWS: Neurological Early Warning Score

Outcome	Minimal risk (≤ 1 point)	Low risk (2-5 points)	Moderate risk (6 points)	High risk (≥ 7 points)	p-value	U-Mann Whitney
In-hospital mortality at hospital admission	0% (0-0%)	1.7% (0-4%)	0% (0-0%)	37.5% (1.6-73.4%)	0.003663	158.5
Urgent transfer to a critical care unit at hospital admission	0% (0-0%)	5% (1.1-9%)	16.7% (0-38.7%)	12.5% (0-37%)	0.028323	468.5
Composite outcome at hospital admission	0% (0-0%)	5% (1.1-9%)	16.7% (0-38.7%)	37.5% (1.6-73.4%)	0.001061	456.5
In-hospital mortality 8h prior to outcome	0% (0-0%)	0.8% (0-2.4%)	0% (0-0%)	40% (8-72%)	0.000213	95.5
Urgent transfer to a critical care unit 8h prior to outcome	0% (0-0%)	4.1% (0.6-7.6%)	11.8% (0-27.6%)	20% (0-46.1%)	0.015096	446.5
Composite outcome 8h prior to outcome	0% (0-0%)	4.1% (0.6-7.6%)	11.8% (0-27.6%)	40% (8-72%)	0.000469	436.5

## Discussion

This study analyzed a homogeneous patient population, thereby reducing the likelihood that specific characteristics or variables introduced bias or negatively affected the results. The median age of patients in our cohort was higher than the national average for hospital admissions in Mexico [24]. Notably, patients with neurological diagnoses exhibited a significantly higher incidence of unplanned transfers to critical care or in-hospital death compared to those with other conditions (61 vs. 22 episodes per 1,000 admissions, respectively). This elevated incidence was consistent across both neurological and neurosurgical groups. While the composite outcome incidence in our cohort aligns with that reported in previous EWS studies, it is markedly higher among patients with neurological disorders. These findings underscore the elevated risk of clinical deterioration in this population and suggest that patients with neurological conditions may derive greater benefit from EWS implementation than those with other diagnoses [16-19].

Among medical neurological patients, cerebrovascular events were the most frequent diagnosis (17.4%), followed by metabolic encephalopathies (11.3%). These results likely reflect the high prevalence of cardiovascular risk factors and metabolic disease within our population, consistent with national health data [24]. In the neurosurgical subgroup, lumbar radiculopathy (15.2%) was the most common diagnosis, followed by craniotomy (10%). These findings point to a predominance of degenerative and traumatic conditions, particularly in the context of an aging population. Gamma Knife radiosurgery for malignant conditions was the third most frequent procedure; when combined with benign indications, it became the second most prevalent neurosurgical intervention, potentially reflecting referral bias in our center. It is important to note that Gamma Knife is a procedure not widely used worldwide; even in referral centers, it is relatively rare - being more common in our center compared to others globally.

Overall, EWS demonstrated lower predictive accuracy for neurological patients than reported in general medical populations [9,11]. This reduced performance may be attributable to the absence of neurological-specific variables in existing scoring systems or to population-specific factors, such as lower baseline oxygen saturation levels related to altitude. One of the theories proposed here gains strength with the model we developed, since - as shown in the results - when neurological-specific variables are added, the predictive power of an EWS improves. Nevertheless, this is only one possible reason why EWS perform less effectively in neurological populations. Further studies are needed to analyze the specific causes of the lower predictive power of EWS in this group.

Among the validated tools, mREMS and NEWS-2 outperformed RAPS and GCS in predicting primary outcomes across all timeframes and subgroups, as well as secondary outcomes. Among the EWS, there was considerable variability between mREMS and NEWS-2 regarding which performed better, depending on several factors, such as the type of population being analyzed at that moment and the timeframe of evaluation. These results should be interpreted with caution, avoiding definitive conclusions or early assumptions about which of these two EWS is superior. For instance, in some cases - such as surgical and non-surgical neurological patients - the initial score showed a better area under the ROC curve with mREMS, while the confidence interval was more favorable with NEWS-2.

One of the possible reasons for mREMS being one of the two better EWS for predicting the analyzed outcomes may be the greater emphasis placed on GCS within the mREMS score, enhancing its discriminatory power in neurologically compromised patients. For this reason, our hypothesis and suggestion is that mREMS may be more appropriate than NEWS-2 or RAPS for use in neurological populations, emphasizing that further studies are needed to reach a definitive conclusion and to make a fully evidence-based recommendation. Importantly, mREMS offers this improved precision without added complexity. These results emphasize the value of tailoring EWS models to specific clinical contexts by integrating or reweighting key variables. This is consistent with previous findings, which demonstrated that the addition of heart rhythm to NEWS-2 significantly improved predictive performance in cardiovascular patients [20].

Our proposed model further supports this concept by showing that the inclusion of easily accessible variables - such as sex, age, or type of neurological condition - can enhance predictive accuracy. These findings highlight the need for context-specific refinement of EWS, particularly for specialized populations such as neurological inpatients. Future research should continue to evaluate existing models in distinct clinical subgroups to identify limitations and refine them accordingly. Additionally, incorporating other routinely available data, such as biochemical markers (e.g., capillary glucose, inflammatory markers) or imaging findings (e.g., CT or MRI results), may further improve predictive accuracy.

The ultimate goal is to implement such a system in real-world clinical settings to reduce morbidity and mortality in neurological patients, in conjunction with RRTs. Should existing EWS models prove insufficiently cost-effective, further optimization through the inclusion of additional easily obtainable clinical variables will be essential.

This study has several limitations. The retrospective design is inherently subject to information bias. Moreover, due to logistical constraints, we were unable to exclude patients who had opted for do-not-resuscitate orders or palliative care pathways from the primary outcome, although these patients constituted a small fraction of the total sample.

## Conclusions

Although NEWS-2 is one of the most widely adopted EWS globally, our findings show it performs poorly in hospitalized patients with neurological conditions. These results underscore the need to evaluate alternative EWS tailored to specific hospital subpopulations with distinct demographic and clinical profiles. In our study, the mREMS system demonstrated higher point estimates for AUC predictive performance for catastrophic events and, in our view, represents a more appropriate tool for neurological inpatients.

Additionally, we propose a new predictive model - NEURO-EWS - specifically designed to detect adverse outcomes in patients with neurological and neurosurgical conditions. This model demonstrated strong predictive ability for in-hospital mortality, urgent transfer to critical care, and the composite of both outcomes, performing comparably to or better than widely used systems such as NEWS-2, mREMS, and RAPS. This makes it a potential new EWS that could be used for these types of patients. However, external validation, enhancement of this new EWS with additional variables, and prospective evaluation are necessary before widespread clinical implementation.

## Appendices

**Table 12:** Goodness-of-Fit and Explanatory Power of the Admission Logistic Regression Model for the Composite Outcome During Hospitalization

Model χ²	p-value	Hosmer-Lemeshow χ²	p-value	Cox and Snell R²	Nagelkerke R²
40.735	0.000013	2.28	0.971	0.078	0.214

**Table 13:** Multivariable Logistic Regression Coefficients for the Admission Model Predicting the Composite Outcome During Hospitalization

Predictor	β coefficient	Standard error	Wald test	p-value	OR (95% CI)
Surgical patient	0.788	0.593	1.763	0.184	2.199 (0.687 - 7.035)
Male non-surgical patient	0.853	0.639	1.784	0.182	2.347 (0.671 - 8.204)
Age ≤39	2.064	1.1	3.524	0.06	7.88 (0.913 - 68.004)
Age ≥60	2.8	1.036	7.3	0.007	16.451 (2.157 - 125.436)
Glasgow Coma Scale (GCS) ≤13	1.974	1.087	3.298	0.069	7.199 (0.855 - 60.596)
HR ≤69	1.045	0.489	4.565	0.033	2.842 (1.09 - 7.409)
HR ≥90	1.842	0.585	9.902	0.002	6.308 (2.003 - 19.864)
Temperature ≤35.9	1.263	0.62	4.147	0.042	3.537 (1.048 - 11.932)
RR ≥20	-0.111	0.476	0.055	0.815	0.895 (0.352 - 2.275)
Need for supplemental O_2_	0.114	0.49	0.054	0.816	1.121 (0.429 - 2.928)
Constant	-6.585	1.18	31.156	0	-

**Table 14:** Goodness-of-Fit and Explanatory Power of the 8-Hour Model for the Composite Outcome Within the Next 8 Hours

Model χ²	p-value	Hosmer-Lemeshow χ²	p-value	Cox and Snell R²	Nagelkerke R²
48.45308	5.13 × 10^-7^	1.328	0.995	0.092	0.253

**Table 15:** Multivariable Logistic Regression Coefficients for the 8-Hour Model Predicting the Composite Outcome Within the Next 8 Hours

Predictor	β coefficient	Standard error	Wald test	p-value	OR (95% CI)
Surgical patient	0.66	0.597	1.224	0.269	1.936 (0.601 - 6.236)
Male non-surgical patient	0.927	0.641	2.089	0.148	2.527 (0.719 - 8.879)
Age ≤39	2.167	1.099	3.892	0.049	8.736 (1.014 - 75.241)
Age ≥60	2.624	1.04	6.371	0.012	13.797 (1.798 - 105.895)
Glasgow Coma Scale (GCS) ≤13	2.137	1.027	4.326	0.038	8.473 (1.131 - 63.467)
HR ≤69	0.719	0.553	1.692	0.193	2.052 (0.695 - 6.064)
HR ≥90	1.535	0.603	6.481	0.011	4.643 (1.424 - 15.14)
Temperature ≤35.9	1.359	0.661	4.221	0.04	3.89 (1.064 - 14.218)
RR ≥20	0.569	0.443	1.653	0.199	1.766 (0.742 - 4.205)
Need for supplemental O_2_	0.627	0.432	2.113	0.146	1.873 (0.804 - 4.364)
Constant	-6.74	1.203	31.383	0	-

## References

[1] Durantez-Fernández C, Polonio-López B, Martín-Conty JL (2022). Comparison of nine early warning scores for identification of short-term mortality in acute neurological disease in the emergency department. J Pers Med.

[2] Schizodimos T, Soulountsi V, Iasonidou C, Kapravelos N (2020). An overview of management of intracranial hypertension in the intensive care unit. J Anesth.

[3] Rhee KJ, Fisher CJ Jr, Willitis NH (1987). The rapid acute physiology score. Am J Emerg Med.

[4] (2022). National Early Warning Score (NEWS) 2: Standardizing the assessment of acute-illness severity in the NHS. https://www.rcp.ac.uk/media/a4ibkkbf/news2-final-report_0_0.pdf.

[5] Melero-Guijarro L, Sanz-García A, Martín-Rodríguez F (2023). Prehospital qSOFA, mSOFA, and NEWS2 performance for sepsis prediction: a prospective, multi-center, cohort study. Front Med (Lausanne).

[6] Rahmatinejad Z, Tohidinezhad F, Rahmatinejad F, Eslami S, Pourmand A, Abu-Hanna A, Reihani H (2021). Internal validation and comparison of the prognostic performance of models based on six emergency scoring systems to predict in-hospital mortality in the emergency department. BMC Emerg Med.

[7] Smith ME, Chiovaro JC, O'Neil M (2014). Early warning system scores for clinical deterioration in hospitalized patients: a systematic review. Ann Am Thorac Soc.

[8] Subbe CP, Kruger M, Rutherford P, Gemmel L (2001). Validation of a modified early warning score in medical admissions. QJM.

[9] Peng L, Luo Z, Liang L (2021). Comparison of the performance of 24 early warning scores with the updated national early warning score (NEWS2) for predicting unplanned intensive care unit admission in postoperative brain tumor patients: a retrospective study at a single center. Med Sci Monit.

[10] Alves Silva LM, Moroço DM, Pintya JP, Miranda CH (2021). Clinical impact of implementing a rapid-response team based on the modified early warning score in wards that offer emergency department support. PLoS One.

[11] Alhmoud B, Bonnici T, Patel R, Melley D, Williams B, Banerjee A (2021). Performance of universal early warning scores in different patient subgroups and clinical settings: a systematic review. BMJ Open.

[12] Miller RT, Nazir N, McDonald T, Cannon CM (2017). The modified rapid emergency medicine score: a novel trauma triage tool to predict in-hospital mortality. Injury.

[13] Forster S, McKeever TM, Shaw D (2022). Effect of implementing the NEWS2 escalation protocol in a large acute NHS trust: a retrospective cohort analysis of mortality, workload and ability of early warning score to predict death within 24 hours. BMJ Open.

[14] Arévalo-Buitrago P, Morales-Cané I, Olivares Luque E, Guler I, Rodríguez-Borrego MA, López-Soto PJ (2021). Predictive power of early-warning scores used in hospital emergency departments: a systematic review and meta-analysis. Emergencias.

[15] Spencer W, Smith J, Date P, de Tonnerre E, Taylor DM (2019). Determination of the best early warning scores to predict clinical outcomes of patients in the emergency department. Emerg Med J.

[16] Liljehult J, Christensen T, Christensen KB (2020). Early prediction of one-year mortality in ischemic and hemorrhagic stroke. J Stroke Cerebrovasc Dis.

[17] Olsson T, Lind L (2003). Comparison of the rapid emergency medicine score and APACHE II in nonsurgical emergency department patients. Acad Emerg Med.

[18] Knoery C, Barlas RS, Vart P (2021). Modified early warning score and risk of mortality after acute stroke. Clin Neurol Neurosurg.

[19] Saposnik G, Kapral MK, Liu Y (2011). IScore: a risk score to predict death early after hospitalization for an acute ischemic stroke. Circulation.

[20] Alhmoud B, Bonnici T, Melley D, Patel R, Banerjee A (2023). Performance of digital early warning score (NEWS2) in a cardiac specialist setting: retrospective cohort study. BMJ Open.

[21] Zhang L, Xu D, Zhang T, Hou W, Yixi L (2021). Correlation between interleukin-6, interleukin-8, and modified early warning score of patients with acute ischemic stroke and their condition and prognosis. Ann Palliat Med.

[22] Wang F, An W, Zhang X (2021). Copeptin combined with national early warning score for predicting survival in elderly critically ill patients at the emergency department. Am J Emerg Med.

[23] Global Burden of Disease Collaborative Network (2021). Global Burden of Disease Study 2019 (GBD 2019) Reference Life Table.

[24] Romero-Martínez M, Cuevas-Nasu L, Gaona-Pineda EB, Shamah-Levy T (2024). Technical note on the 2023 continuous national health and nutrition survey: fieldwork results. Salud Publica Mex.

